# Estimation of Distribution Overlap of Urn Models

**DOI:** 10.1371/journal.pone.0042368

**Published:** 2012-11-06

**Authors:** Jerrad Hampton, Manuel E. Lladser

**Affiliations:** Department of Applied Mathematics, University of Colorado, Boulder, Colorado, United States of America; Pennsylvania State University, United States of America

## Abstract

A classical problem in statistics is estimating the expected coverage of a sample, which has had applications in gene expression, microbial ecology, optimization, and even numismatics. Here we consider a related extension of this problem to random samples of two discrete distributions. Specifically, we estimate what we call the dissimilarity probability of a sample, i.e., the probability of a draw from one distribution not being observed in 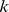 draws from another distribution. We show our estimator of dissimilarity to be a 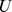-statistic and a uniformly minimum variance unbiased estimator of dissimilarity over the largest appropriate range of 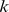. Furthermore, despite the non-Markovian nature of our estimator when applied sequentially over 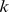, we show it converges uniformly in probability to the dissimilarity parameter, and we present criteria when it is approximately normally distributed and admits a consistent jackknife estimator of its variance. As proof of concept, we analyze V35 16S rRNA data to discern between various microbial environments. Other potential applications concern any situation where dissimilarity of two discrete distributions may be of interest. For instance, in SELEX experiments, each urn could represent a random RNA pool and each draw a possible solution to a particular binding site problem over that pool. The dissimilarity of these pools is then related to the probability of finding binding site solutions in one pool that are absent in the other.

## Introduction

An inescapable problem in microbial ecology is that a sample from an environment typically does not observe all species present in that environment. In [14], this problem has 1been recently linked to the concepts of *coverage probability* (i.e. the probability that a member from the environment is represented in the sample) and the closely related *discovery* or *unobserved probability* (i.e. the probability that a previously unobserved species is seen with another random observation from that environment). The mathematical treatment of coverage is not limited, however, to microbial ecology and has found applications in varied contexts, including gene expression, microbial ecology, optimization, and even numismatics.

The point estimation of coverage and discovery probability seem to have been first addressed by Turing and Good [2] to help decipher the Enigma Code, and subsequent work has provided point predictors and prediction intervals for these quantities under various assumptions [1], [3]–[5].

Following Robbins [6] and in more generality Starr [7], an unbiased estimator of the expected discovery probability of a sample of size 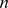 is
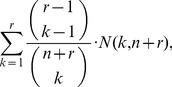
(1)where 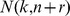 is the number of species observed exactly 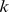-times in a sample with replacement of size 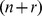. Using the theory of U-statistics developed by Halmos [8], Clayton and Frees [9] show that the above estimator is the *uniformly minimum variance unbiased estimator* (UMVUE) of the expected discovery probability of a sample of size 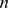 based on an enlarged sample of size 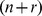.

A quantity analogous to the discovery probability of a sample from a single environment but in the context of two environments is *dissimilarity*, which we broadly define as the probability that a draw in one environment is not represented in a random sample (of a given size) from a possibly different environment. Estimating the dissimilarity of two microbial environments is therefore closely related to the problem of assessing the species that are unique to each environment, and the concept of dissimilarity may find applications to measure sample quality and allocate additional sampling resources, for example, for a more robust and reliable estimation of the UniFrac distance [10], [11] between pairs of environments. Dissimilarity may find applications in other and very different contexts. For instance, in SELEX experiments [12]–a laboratory technique in which an initial pool of synthesized random RNA sequences is repeatedly screened to yield a pool containing only sequences with given biological functions–the dissimilarity of two RNA pools corresponds to the probability of finding binding site solutions in one pool that are absent in the other.

In this manuscript, we study an estimator of dissimilarity probability similar to Robbins' and Starr's statistic for discovery probability. Our estimator is optimal among the appropriate class of unbiased statistics, while being approximately normally distributed in a general case. The variance of this statistic is estimated using a consistent jackknife. As proof of concept, we analyze samples of processed V35 16S rRNA data from the Human Microbiome Project [13].

### Probabilistic Formulation and Inference Problem

To study dissimilarity probability, we use the mathematical model of a pair of urns, where each urn has an unknown composition of balls of different colors, and where there is no a priori knowledge of the contents of either urn. Information concerning the urn composition is inferred from repeated draws with replacement from that urn.

In what follows, 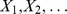 and 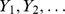 are independent sequences of independent and identically distributed (i.i.d.) discrete random variables with probability mass functions 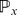 and 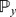, respectively. Without loss of generality we assume that 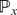 and 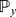 are supported over possibly infinite subsets of 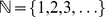, and think of outcomes from these distributions as “colors”: i.e. we speak of color-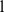, color-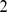, etc. Let 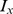 denote the set of colors 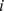 such that 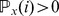, and similarly define 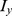. Under this perspective, 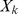 denotes the color of the 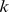-th ball drawn with replacement from urn-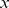. Similarly, 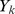 is the color of the 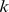-th ball drawn with replacement from urn-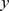. Note that based on our formulation, distinct draws are always independent.

The mathematical analysis that follows was motivated by the problem of estimating the fraction of balls in urn-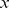 with a color that is absent in urn-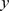. We can write this parameter as
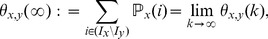
(2)where



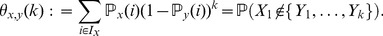
(3)The parameter 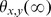 measures the proportion of urn-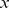 which is unique from urn-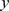. On the other hand, 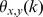 is a measure of the effectiveness of 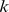-samples from urn-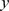 to determine uniqueness in urn-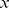. This motivates us to refer to the quantity in (2) as the *dissimilarity of urn-* 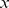 *from urn-* 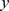, and to the quantity in (3) as the *average dissimilarity of urn-* 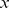 *relative to* 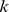 *-draws from urn-* 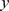. Note that these parameters are in general asymmetric in the roles of the urns. In what follows, urns-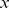 and -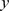 are assumed fixed, which motivates us to remove subscripts and write 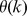 instead of 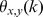.

Unfortunately, one cannot estimate unbiasedly the dissimilarity of one urn from another based on finite samples, as stated in the following result. (See the Materials and Methods section for the proofs of all of our results).

**Theorem 1** *(No unbiased estimator of dissimilarity.) There is no unbiased estimator of 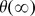 based on finite samples from two arbitrary urns-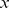 and -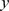.*

Furthermore, estimating 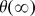 accurately without further assumptions on the compositions of urns-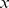 and -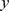 seems a difficult if not impossible task. For instance, arbitrarily small perturbations of urn-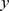 are likely to be unnoticed in a sample of a given size from this urn but may drastically affect the dissimilarity of other urns from urn-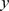. To demonstrate this idea, consider a parameter 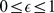 and let 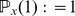, 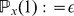 and 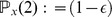. If 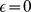 then 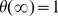 while, for each 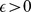, 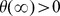.

In contrast with the above, for fixed 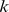, 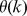 depends continuously on 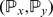 e.g. under the metric
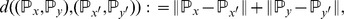
where 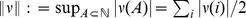 denotes the total variation of a signed measure 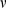 over 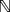 such that 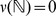. This is the case because



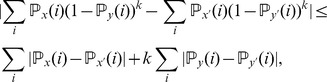


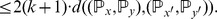



The above implies that 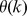 is continuous with respect to any metric equivalent to 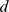. Many such metrics can be conceived. For instance, if 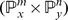 denotes the probability measure associated with 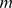 samples with replacement from urn-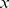 that are independent of 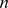 samples with replacement from urn-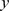 then 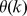 is also continuous with respect to any of the metrics 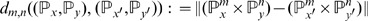, with 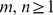, because


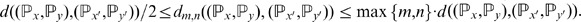
Because of the above considerations, we discourage the direct estimation of 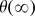 and focus on the problem of estimating 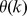 accurately.

## Results

Consider a finite number of draws with replacement 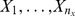 and 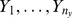, from urn-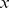 and urn-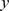, respectively, where 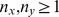 are assumed fixed. Using this data we can estimate 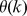, for 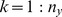, via the estimator:
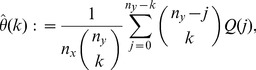
(4)where



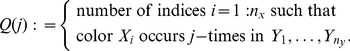
(5)We refer to 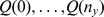 as the 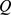-statistics summarizing the data from both urns. Due to the well-known relation: 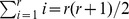, at most 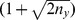 of these estimators are non-zero. This sparsity may be exploited in the calculation of the right-hand side of (4) over a large range of 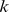
's.

Our statistic in 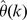 is the U-statistic associated with the kernel 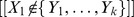, where 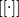 is used to denote the indicator function of the event within the brackets (Iverson's bracket notation). Following the approach by Halmos in [8], we can show that this U-statistic is optimal amongst the unbiased estimators of 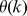 for 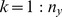. We note that no additional samples from either urn are necessary to estimate 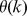 unbiasedly over this range when 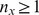. This contrasts with the estimator in equation (1), which requires sample enlargement for unbiased estimation of discovery probability of a sample of size 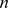.

**Theorem 2** *(Minimum variance unbiased estimator.) If* 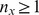 *and* 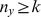 *then* 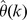 *is the unique uniformly minimum variance unbiased estimator of* 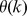 *. Further, no unbiased estimator of* 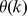 *exists for* 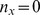 *or* 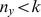.

Our next result shows that 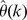 converges uniformly in probability to 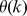 over the largest possible range where unbiased estimation of the later parameter is possible, despite the non-Markovian nature of 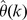 when applied sequentially over 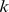. The result asserts that 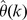 is likely to be a good approximation of 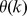, uniformly for 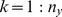, when 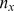 and 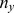 are large. The method of proof uses an approach by Hoeffding [14] for the exact calculation of the variance of a 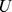-statistic.

**Theorem 3** *(Uniform convergence in probability.) Independently of* *how* 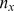 *and* 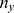 *tend to infinity, it follows for each 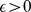 that*


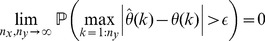
(6)

We may estimate the variance of 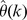 for 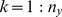 via a leave-one-out or also called delete-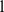 jackknife estimator, using an approach studied by Efron and Stein [15] and Shao and Wu [16].

To account for variability in the 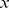-data through a leave-one-out jackknife estimate, we require that 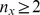 and let
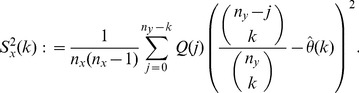
(7)


On the other hand, to account for variability in the 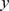-data, consider for 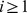 and 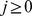 the statistics
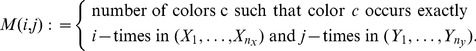
(8)


Clearly, 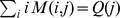; in particular, the 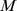-statistics are a refinement of the 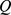-statistics. Define 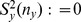 and, for 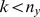, define
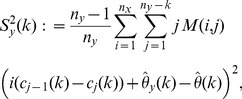
(9)where



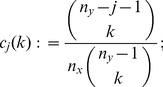
(10)

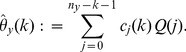
(11)


Our estimator of the variance of 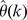 is obtained by summing the variance attributable to the 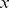-data and the 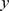-data and is given by
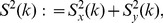
(12)for 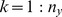; in particular, 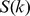 is our jackknife estimate of the standard deviation of 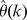.

To assess the quality of 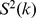 as an estimate of the variance of 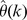 and the asymptotic distribution of the later statistic, we require a few assumptions that rule out degenerate cases. The following conditions are used in the remaining theorems in this section:

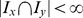
.
- there are at least two colors in 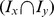 that occur in different proportions in urn-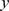; in particular, the conditional probability 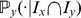 is not a uniform distribution.
- urn-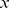 contains at least one color that is absent in urn-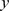; in particular, 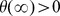.

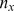
 and 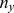 grow to infinity at a comparable rate i.e. 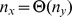, which means that there exist finite constants 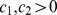 such that 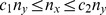, as 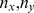 tend to infinity.

Conditions (a–c) imply that 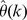 has a strictly positive variance and that a projection random variable, intermediate between 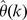 and 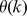, has also a strictly positive variance. The idea of projection is motivated by the analysis of Grams and Serfling in [17].

Condition (d) is technical and only used to show that the result in Theorem 5 holds for the largest possible range of values of 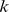 namely, for 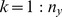. See [18] for results with uniformity related to Theorem 4, as well as uniformity results when condition (d) is not assumed.

Because the variance of 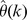, from now on denoted 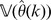, and its estimate 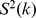 tend to zero as 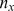 and 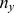 increase, the unnormalized consistency result is unsatisfactory. As an alternative, we can show that 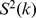 is a consistent estimator relative to 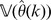, as stated next.

**Theorem 4** *(Asymptotic consistency of variance estimation.) If conditions (a)–(c) are satisfied then, for each* 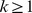 *and 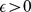, it applies that*


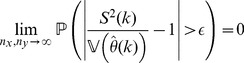
(13)

Finally, under conditions (a)–(d), we show that 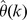 is asymptotically normally distributed for all 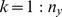, as 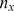 and 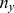 increase at a comparable rate.

**Theorem 5** *(Asymptotic normality.) Let* 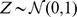 *i.e.* 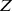 *has a standard normal distribution. If conditions (a)–(d) are satisfied then*

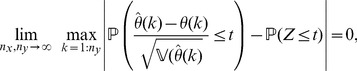
(14)
*for all real number* 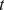.

The non-trivial aspect of the above result is the asymptotic normality of 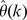 when 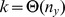, e.g. 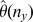, as the results we have found in the literature [14],[19],[20] only guarantee the asymptotic normality of our estimator of 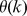 for fixed 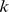. We note that, due to Slutsky's theorem [21], it follows from (13) and (14) that the ratio
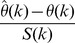
has, for fixed 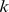, approximately a standard normal distribution when 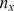 and 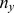 are large and of a comparable order of magnitude.

## Discussion

As proof of concept, we use our estimators to analyze data from the Human Microbiome Project (HMP) [13]. In particular, our samples are V35 16S rRNA data, processed by Qiime into an operational taxonomic unit (OTU) count table format (see File S1). Each of the 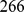 samples analyzed have more than 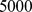 successfully identified bacteria (see File S2). We sort these samples by the body location metadata describing the origin of the sample. This sorting yields the assignments displayed in Table 1.

**Table 1:** HMP data.

Body Supersite	Body Subsite	Assigned Labels
Airways	Anterior Nares	1–5
	Throat	6–17
Gastrointestinal Tract	Stool	18–47
Oral	Attached/Keratinized Gingiva	48–59
	Buccal Mucosa	60–76
	Hard Palate	77–90
	Palatine Tonsils	91–112
	Saliva	113–122
	Subgingival Plaque	123–144
	Supragingival Plaque	145–167
	Tongue Dorsum	168–191
Skin	Left Antecubital Fossa	192–195
	Left Retroauricular Crease	196–217
	Right Antecubital Fossa	218–222
	Right Retroauricular Crease	223–242
Urogenital Tract	Mid Vagina	243–248
	Posterior Fornix	249–259
	Vaginal Introitus	260–266

Summary of V35 16S rRNA data processed by Qiime into an OTU table.

**Figure 1:**
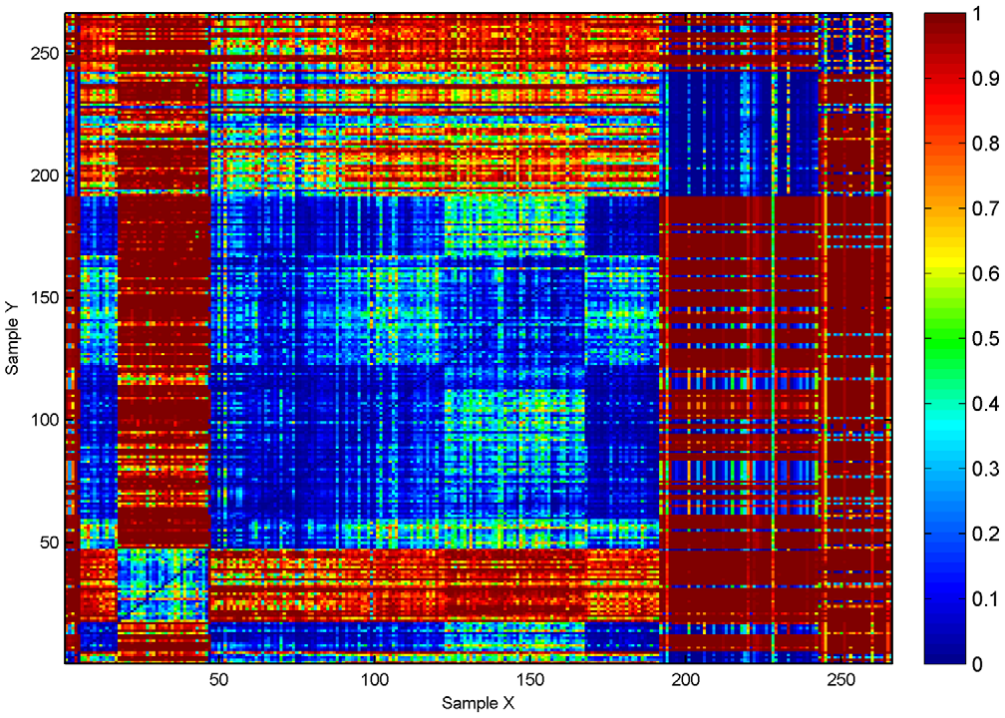
Dissimilarity estimates. Heat map of 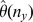 sorted by site location metadata. Here, the 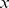-axis denotes the sample from the environment corresponding to urn-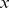, and similarly for the 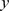-axis. The entries on the diagonal are set to zero.

We present our estimates of 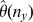 for all 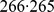 possible sample comparisons in Figure 1, i.e., we estimate the average dissimilarity of sample-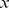 relative to the full sample-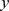. Due to (4), observe that 
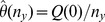
. At the given sample sizes, we can differentiate four broad groups of environments: stool, vagina, oral/throat and skin/nostril. We differentiate a larger proportion of oral/throat bacteria found in stool than stool bacteria found in the oral/throat environments. We may also differentiate the throat, gingival and saliva samples, but cannot reliably differentiate between tongue and throat samples or between the subgingival and supragingival plaques. On the other hand, the stool samples have larger proportions of unique bacteria relative to other stool samples of the same type, and vaginal samples also have this property. In contrast the skin/nostril samples have relatively few bacteria that are not identified in other skin/nostril samples.

**Figure 2:**
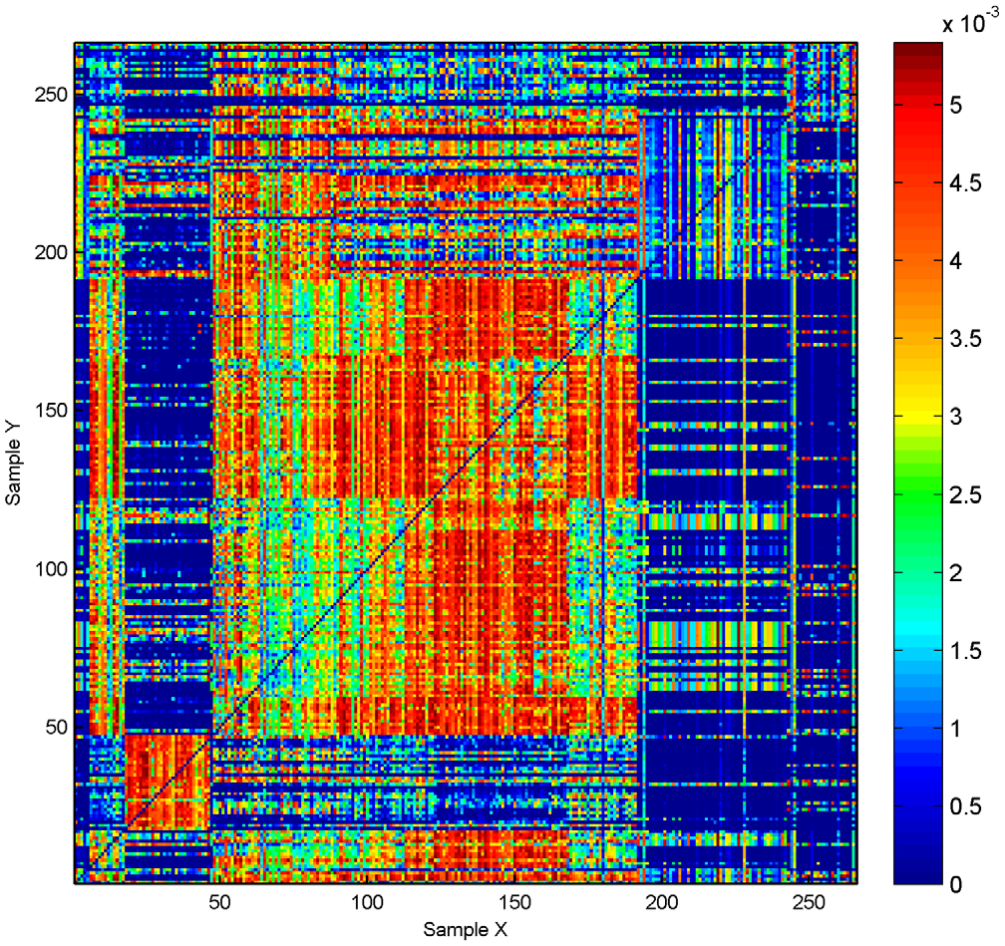
Error estimates. Heat map of 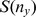 sorted by site location metadata. Here, the 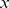-axis also denotes the sample from the environment corresponding to urn-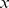, and similarly for the 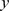-axis, and the entries on the diagonal are again set to zero.



The above effects may be a property of the environments from which samples are taken, or an effect of noise from inaccurate estimates due to sampling. To rule out the later interpretation, we show estimates of the standard deviation of 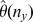 based on the jackknife estimator 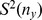 from (12) in Figure 2. As 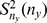 is zero, the error estimate is given by 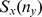. We see from (7), with 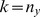, that

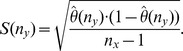



Assuming a normal distribution and an accurate jackknife estimate of variance, 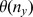 will be in the interval 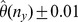 with at least approximately 95% confidence, for any choice of sample comparisons in our data; in particular, on a linear scale, we expect at least 95% of the estimates in Figure 1 to be accurate in at least the first two digits.

As we mentioned earlier, estimating 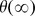 accurately is a difficult problem. We end this section with two heuristics to assess how representative 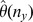 is of 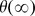, when urn-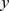 has at least two colors and at least one color in common with urn-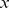. First, observe that:
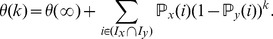
(15)


In particular, 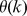 is a strictly concave-up and monotonically decreasing function of the real-variable 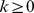. Hence, if 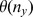 is close to the asymptotic value 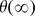, then 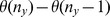 should be of small magnitude. We call the later quantity the *discrete derivative* of 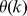 at 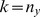. Since we may estimate the discrete derivative from our data, the following heuristic arises: *relatively large values of* 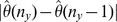 *are evidence that* 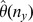 *is not a good approximation of* 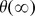.

**Figure 3:**
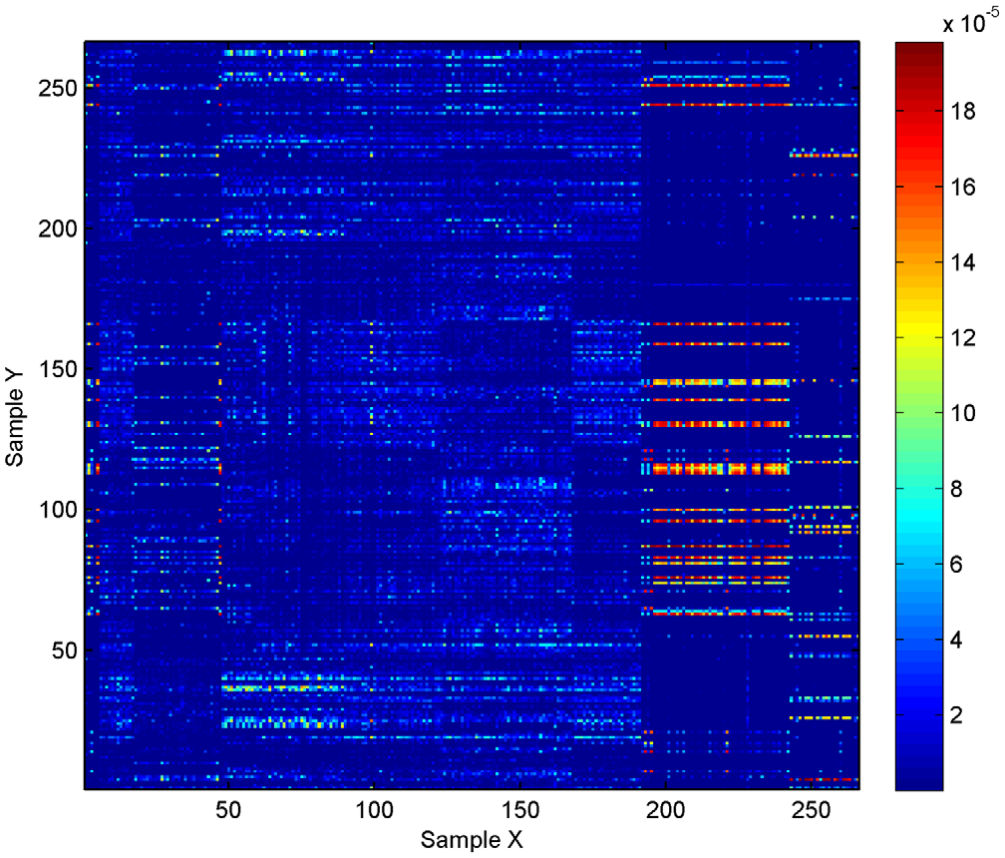
Discrete derivative estimates. Heat map of 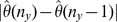, sorted by site location metadata, following the same conventions as in the previous figures.

Figure 3 shows the heat map of 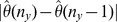 for each pair of samples. These estimates are of order 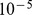 for the majority of the comparisons, and spike to 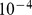 for several sample-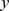 of varied environment types, when sample-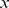 is associated with a skin or vaginal sample. In particular, further sampling effort from environments associated with certain vaginal, oral or stool samples are likely to reveal bacteria associated with broadly defined skin or vaginal environments.

Another heuristic may be more useful to assess how close 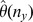 is to 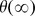, particularly when the previous heuristic is inconclusive. As motivation, observe that 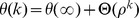, because of the identity in (15), where
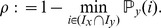



Furthermore, 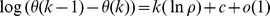, where 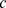 is certain finite constant. We can justify this approximation only when 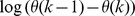 is well approximated by a linear function of 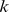, in which case we let 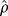 denote the estimated value for 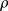 obtained from the linear regression. Since 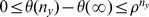, the following more precise heuristic comes to light: 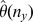 *is a good approximation of* 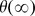 *if the linear regression of* 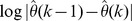 *for* 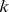 *near* 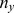 *gives a good fit,* 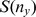 *is small relative to* 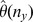 *, and* 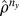 *is also small.*

To fix ideas we have applied the above heuristic to three pairs of samples: 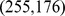, 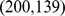 and 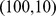, with each ordered pair denoting urn-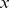 and urn-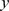, respectively. As seen in Table 2 for these three cases, 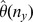 is at least 14-times larger than 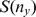; in particular, due to the asymptotic normality of the later statistic, an appropriate use of the heuristic is reduced to a good linear fit and a small 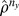 value. In all three cases, 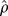 was computed from the estimates 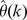, with 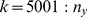.

**Table 2:** Sample comparisons.

Urn- 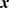	Urn- 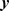	n*_χ_*	n*_y_*	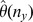	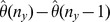	Regression Error	*S*(*n_y_*)	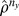
255	176	5054	6782	0.9998	0.0	0.0	1.9892×10^−4^	0.0
200	139	12747	5739	0.0499	−1.6533×10^−4^	6.8306×10^−6^	1.9286×10^−3^	0.9997
100	10	6206	8655	0.0324	−2.9416×10^−6^	0.0438	2.2477×10^−3^	0.5130

Summary of estimates for three pairs of samples of the HMP data.

**Figure 4:**
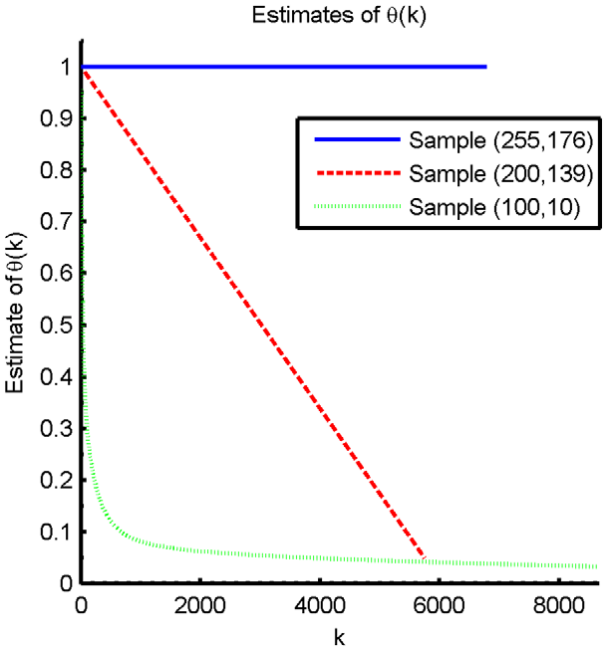
Sequential estimation. Plots of 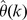, with 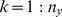, for three pairs of samples of the HMP data.

For the 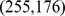-pair, 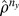 and the regression error, measured as the largest absolute residual associated with the best linear fit, are zero to machine precision, suggesting that 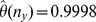 is a good approximation of 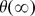. This is reinforced by the blue plot in Figure 4. On the other hand, for the 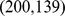-pair, the regression error is small, suggesting that the linear approximation 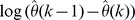 is good for 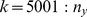. However, because 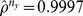, we cannot guarantee that 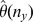 is a good approximation of 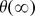. In fact, as seen in the red-plot in Figure 4, 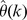, with 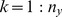, exposes a steady and almost linear decay that suggests that 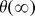 may be much smaller than 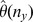. Finally, for the 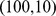-pair, the regression error is large and the heuristic is therefore inconclusive. Due to the green-plot in Figure 4, the lack of fit indicates that the exponential rate of decay of 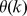 to 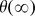 has not yet been captured by the data from these urns. Note that the heuristic based on the discrete derivative shows no evidence that 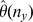 is far from 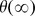.

## Materials and Methods

Here we prove the theorems given in the Results section. The key idea to prove each theorem may be summarized as follows.

To show Theorem 1, we identify pairs of urns for which unbiased estimation of 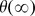 is impossible for any statistic. To show Theorem 2, we exploit the diversity of possible urn distributions to show that there are relatively few unbiased estimators of 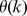 and, in fact, there is a single unbiased estimator 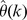 that is symmetric on the data. The uniqueness of the symmetric estimator is obtained via a completeness argument: a symmetric statistic having expected value zero is shown to correspond to a polynomial with identically zero coefficients, which themselves correspond to values returned by the statistic when presented with specific data. The symmetric estimator is a U-statistic in that it corresponds to an average of unbiased estimates of 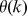, based on all possible sub-samples of size 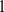 and 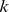 from the samples of urn-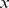 and -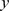, respectively. As any asymmetric estimator has higher variance than a corresponding symmetric estimator, the symmetric estimator must be the UMVUE.

To show Theorem 3 we use bounds on the variance of the U-statistic and show that, uniformly for relatively small 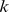, 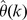 converges to 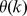 in the 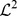-norm. In contrast, for relatively large values of 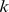, we exploit the monotonicity of 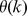 and 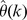 to show uniform convergence.

Finally, theorems 4 and 5 are shown using an approximation of 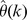 by sums i.i.d. random variables, as well as results concerning the variance of both 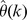 and its approximation. In particular, the approximation satisfies the hypotheses the Central Limit Theorem and Law of Large Numbers, which we use to transfer these results to 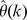.

In what follows, 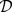 denotes the set of all probability distributions that are finitely supported over 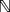.

### Proof of Theorem 1

Consider in 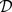 probability distributions of the form 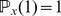, 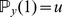 and 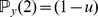, where 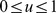 is a given parameter. Any statistic 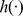 which takes as input 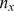 draws from urn-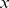 and 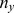 draws from urn-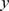 has that 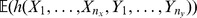 is a polynomial of degree at most 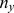 in the variable 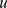; in particular, it is a continuous function of 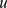 over the interval 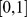. Since 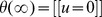 has a discontinuity at 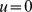 over this interval, there exists no estimator of 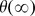 that is unbiased over pairs of distributions in 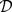.

We use lemmas 6–11 to first show Theorem 2. The method of proof of this theorem follows an approach similar to the one used by Halmos [8] for single distributions, which we extend here naturally to the setting of two distributions.

Our next result implies that no uniformly unbiased estimator of 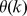 is possible when using less than one sample from urn-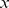 and 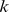 samples from urn-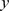.

**Lemma 6** *If* 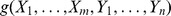 *is unbiased for* 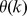 *for all* 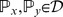 *, then* 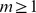 *and* 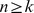.

*Proof*. Consider in 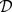 probability distributions of the form 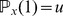, 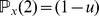, 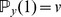 and 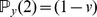, where 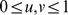 are arbitrary real numbers. Clearly, 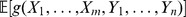 is a linear combination of polynomials of degree 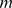 in 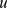 and 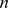 in 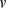 and, as a result, it is a polynomial of degree at most 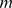 in 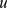 and 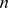 in 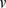. Since 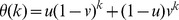 has degree 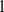 in 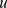 and 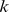 in 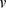, and 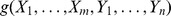 is unbiased for 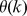, we conclude that 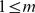 and 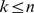.

The form of 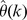 given in equation (4) is convenient for computation but, for mathematical analysis, we prefer its 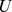-statistic form associated with the kernel function 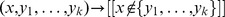.

In what follows, 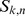 denotes the set of all functions 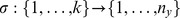 that are one-to-one.

**Lemma 7**

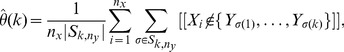
(16)
*where* 
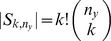
.

*Proof*. Fix 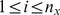 and suppose that color 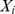 occurs 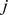-times in 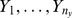. If 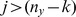 then any sublist of size 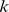 of 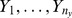 contains 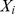, hence 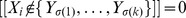, for all 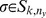. On the other hand, if 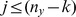 then 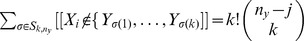. Since the rightmost sum only depends on the number of times that color 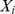 was observed in 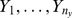, we may use the 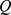-statistics defined in equation (5) to rewrite:
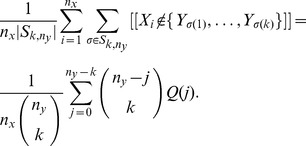



The right-hand side above now corresponds to the definition of 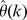 given in equation (4).

In what follows, we say that a function 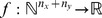 is 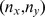 *-symmetric* when
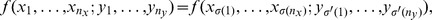
for all 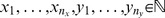 and permutations 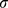 and 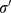 of 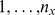 and 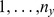, respectively. Alternatively, 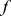 is 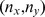-symmetric if and only if it may be regarded a function of 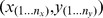, where 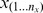 and 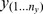 correspond to the order statistics 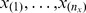 and 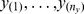, respectively. Accordingly, a statistic of 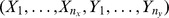 is called 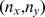 *-symmetric* when it may be represented in the form 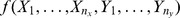, for some 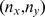-symmetric function 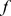. It is immediate from Lemma 7 that 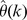 is 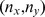-symmetric.

The next result asserts that the variance of any non-symmetric unbiased estimator of 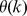 may be reduced by a corresponding symmetric unbiased estimator. The proof is based on the well-known fact that conditioning preserves the mean of a statistic and cannot increase its variance.

**Lemma 8** *An asymmetric unbiased estimator of* 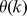 *that is square-integrable has a strictly larger variance than a corresponding* 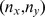 *-symmetric unbiased estimator.*

*Proof*. Let 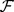 denote the sigma-field generated by the random vector 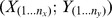 and suppose that the statistic 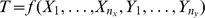 is unbiased for 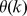 and square-integrable. In particular, 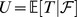 is a well-defined statistic and there is an 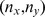-symmetric function 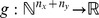 such that 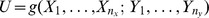. Clearly, 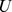 is unbiased for 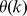 and 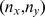-symmetric. Since 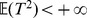, Jensen's inequality for conditional expectations [22] implies that 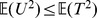, with equality if and only if 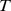 is 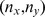-symmetric.

Since 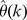 is 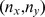-symmetric and bounded, the above lemma implies that if an UMVUE for 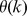 exists then it must be 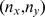-symmetric. Next, we show that there is a unique symmetric and unbiased estimator of 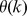, which immediately implies that 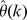 is the UMVUE.

In what follows, 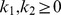 denote integers. We say that a polynomial 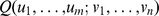 is 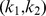 *-homogeneous* when it is a linear combination of polynomials of the form 
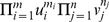
, with 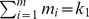 and 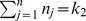. Furthermore, we say that 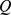 satisfies the *partial vanishing condition* if 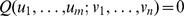 whenever 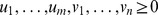, 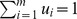 and 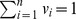.

The next lemma is an intermediate step to show that a 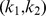-homogeneous polynomial which satisfies the partial vanishing condition is the zero polynomial, which is shown in Lemma 10.

**Lemma 9** *If* 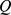 *is a* 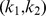 *-homogeneous polynomial in the real variables* 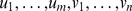 *, with* 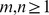 *, that satisfies the partial vanishing condition, then* 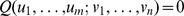 *whenever* 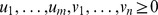 *,* 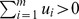 *and* 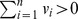.

*Proof*. Fix 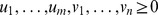 such that 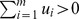 and 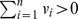 and observe that
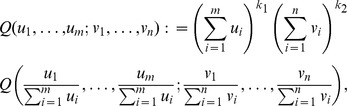
because 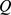 is a 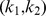-homogeneous polynomial. Notice now that the right hand-side above is zero because 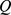 satisfies the partial vanishing condition.

**Lemma 10** *Let* 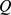 *be a* 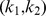 *-homogeneous polynomial in the real variables* 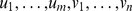 *, with* 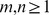 *. If* 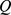 *satisfies the partial vanishing condition then* 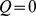 *identically.*

*Proof*. We prove the lemma using structural induction on 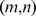 for all 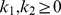.

If 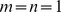 then a 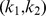-homogeneous polynomial 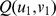 must be of the form 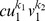, for an appropriate constant 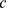. As such a polynomial satisfies the partial-vanishing condition only when 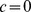, the base case for induction is established.

Next, consider a 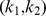-homogeneous polynomial 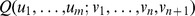, with 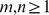, that satisfies the partial vanishing condition, and let 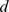 denote its degree with respect to the variable 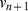. In particular, there are polynomials 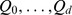 in the variables 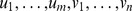 such that
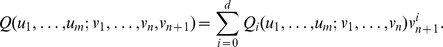



Now fix 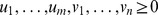 such that 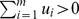 and 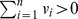. Because 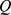 satisfies the partial vanishing condition, Lemma 9 implies that 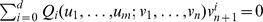 for all 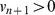. In particular, for each 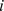, 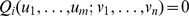 whenever 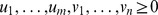, 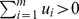 and 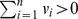. Thus each 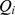 satisfies the partial vanishing condition. Since 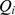 is a 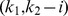-homogeneous polynomial, the inductive hypothesis implies that 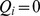 identically and hence 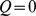 identically. The same argument shows that if 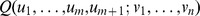, with 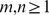, is a 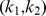-homogeneous polynomial that satisfies the partial vanishing condition then 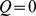 identically, completing the inductive proof of the lemma.

Our final resultbefore proving Theorem 2 implies that 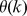 cannot admit more than one symmetric and unbiased estimator. Its proof depends on the variety of distributions in 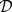, and uses the requirement that our estimator must be unbiased for any pair of distributions chosen from 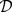.

**Lemma 11** *If* 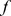 *is an* 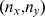 *-symmetric function such that* 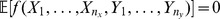, for all 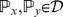, *then* 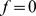 *identically.*

*Proof*. Consider a point 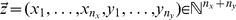 and define 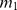 and 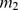 as the cardinalities of the sets 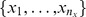 and 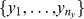, respectively. Furthermore, let 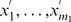 denote the distinct elements in the set 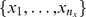 and define 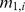 to be the number of times that 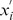 appears in this set. Furthermore, let 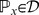 be a probability distribution such that 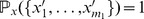 and define 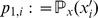. In a completely analogous manner define 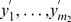, 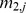, 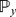 and 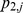.

Notice that 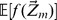 is a polynomial in the real variables 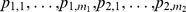 that satisfies the hypothesis of Lemma 10; in particular, this polynomial is identically zero. However, because 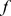 is 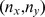-symmetric, the coefficient of 
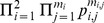
 in 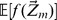 is
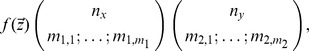
implying that 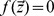.

### Proof of Theorem 2

From Lemma 8, as we mentioned already, if the UMVUE for 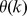 exists then it must be 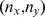-symmetric. Suppose there are two 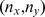-symmetric functions such that 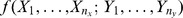 and 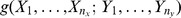 are unbiased for 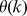. Applying Lemma 11 to 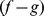 shows that 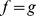, and 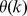 admits therefore a unique symmetric and unbiased estimator. From Lemma 7, 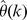 is 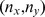-symmetric and unbiased for 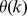 hence it is the UMVUE for 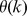. From Lemma 6, it follows that no unbiased estimator of 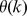 exists for 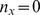 or 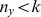.

Our next goal is to show Theorem 3, for which we prove first lemmas 12–13. We note that the later lemma applies in a much more general context than our treatment of dissimilarity.

**Lemma 12** *If, for each* 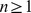 *,* 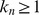 *is an integer such that* 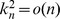 *then*

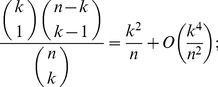
(17)

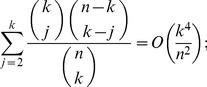
(18)
*uniformly for* 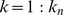 *as* 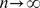.

*Proof*. First observe that for all 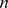 sufficiently large and 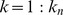, it applies that
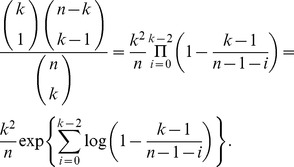



Note that 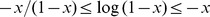, for all 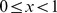. As a result, we may bound the exponential factor on the right-hand side above as follows:
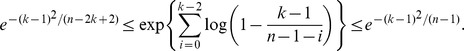



Since 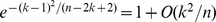 and 
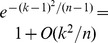
, uniformly for all 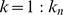 as 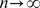, (17) follows.

To show (18), first note the combinatorial identity
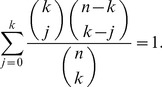
(19)


Proceeding in an analogous manner as we did to show (17), we see now that the term associated with the index 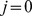 in the above summation satisfies that
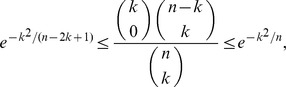
for all 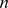 sufficiently large and 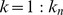. Since 
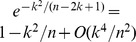
 and 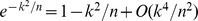, the above inequalities together with (17) and (19) establish (18).

**Lemma 13** *Define* 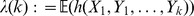 *, where* 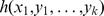 *is a bounded* 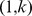 *-symmetric function, and let*

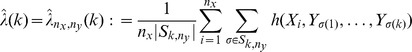

*be the U-statistic of* 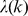 *associated with* 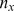 *draws from urn*-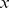 *and* 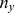 *draws from urn*-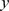; *in particular*, 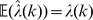. *Furthermore, assume that*

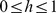
,
- *there is a function* 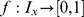 *such that 
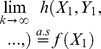
*,

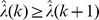
; *in particular*, 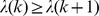.

*Under the above assumptions, it follows that*

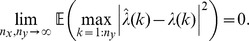



*Proof*. Define 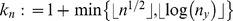 ; in particular, 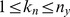 and 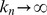, 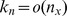 and 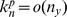, for any 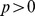, as 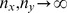. The proof of the theorem is reduced to show that
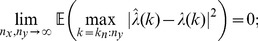
(20)

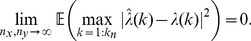
(21)


Next, we compute the variance of 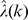 following an approach similar to Hoeffding [14]. Because 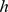 is 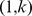-symmetric, a tedious yet standard calculation shows that
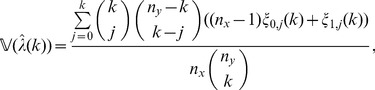
(22)where



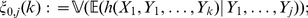
(23)

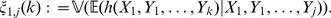
(24)


Clearly, 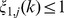. On the other hand, if 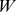 is any random variable with finite expectation and 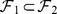 are sigma-fields then 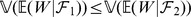, due to well-known properties of conditional expectations [22]. In particular, for each 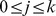, we have that
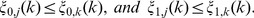
(25)


Consequently, (22) implies that
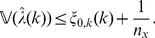
(26)


We claim that
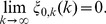
(27)


Indeed, using an argument similar as above, we find that
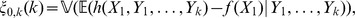


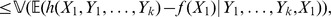


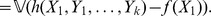



Due to assumptions (*i*)-(*ii*) and the Bounded Convergence Theorem, the right-hand side above tends to 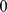, and the claim follows.

It follows from (26) and (27) that
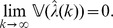



Finally, because of assumption (*iii*),
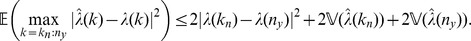



Since each term on the right-hand side above tends to zero as 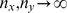, (20) follows.

We now show (21). As 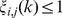 and 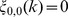, it follows by (22) and Lemma 12 that
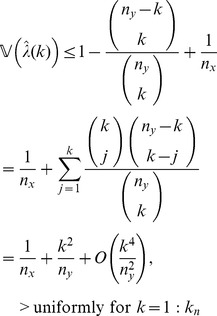
uniformly for 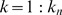 as 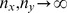. In particular,



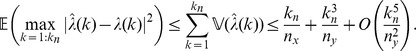



Due to the definition of the coefficients 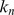, the right-hand side above tends to zero, and (21) follows.

### Proof of Theorem 3

Note that 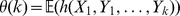, with 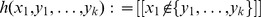. We show that the kernel function 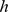 and the U-statistics 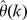 satisfy the hypotheses of Lemma 13. From this the theorem is immediate because 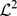-convergence implies convergence in probability.

Clearly 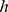 is 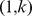-symmetric and 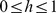, which shows assumption (i) in Lemma 13. On the other hand, due to the Law of Large Numbers, 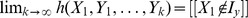 almost surely, from which assumption (ii) in the lemma also follows.

Finally, to show assumption (iii), recall that 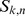 is the set of one-to-one functions from 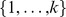 into 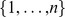; in particular, 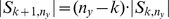. Now note that for each indicator of the form 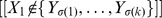, with 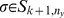, there are 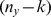 choices of 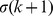 outside the set 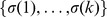. Because 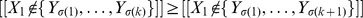, it follows that 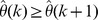 for all 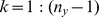. This shows condition (iii) in Lemma 13, and Theorem 3 follows.

### Proof of equation (7)

The jackknife estimate of the variance of 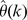 obtained from removing a single 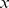-data is, by definition, the quantity
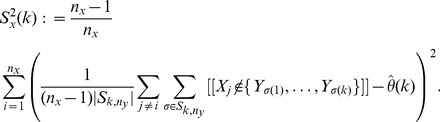
(28)


Note that removing a color from the 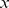-data which would otherwise add to 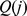, decrements this quantity by one unit. Let 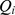 denote the 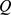-statistics associated with the data when observation 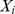 from urn-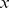 is removed from the sample. Note that as each draw from urn-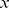 contributes to exactly one 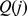, 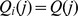 for all 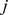 except for some 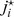 where 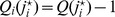. We have therefore that
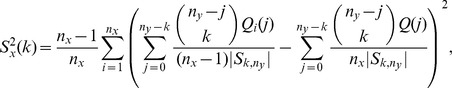


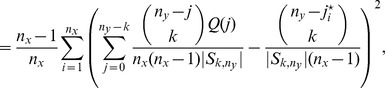


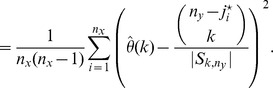



Since there are 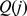 draws from urn-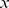 which contribute to 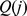, the above sum may be now rewritten in the form given in (7).

### Proof of equation (9)

Similarly, 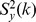 corresponds to the jackknife summed over each possible deletion of a single 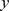-data, which is more precisely given by
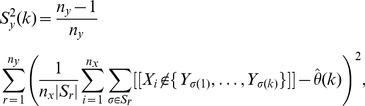
(29)where 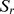 is the set of one-to-one functions from 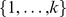 into 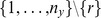.

Recall that 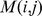 is the number of colors seen 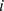 times in draws from urn-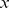 and 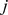 times in draws from urn-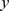, giving that 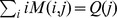.

Fix 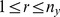 and suppose that 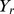 is of a color that contributes to 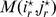, for some 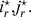 Removing 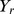 from the data decrements 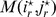 and increments 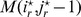 by one unit. Proceeding similarly as in the case for 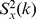, if 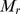 is used to denote the 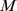-statistics when observation 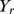 is removed from sample-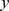, then
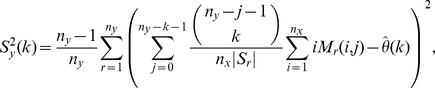


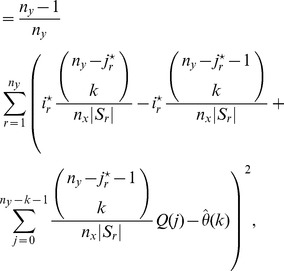


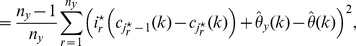
where 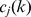 and 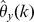 are as defined in (10) and (11). Noting that for each 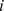 there are 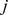 draws from urn-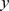 that contribute to 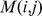, the form in (9) follows.

In what follows, we specialize the coefficients in (23) and (24) to the kernel function of dissimilarity, 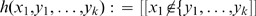. From now on, for each 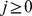 and 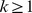, define
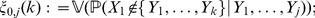
(30)

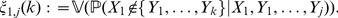
(31)


Above it is understood that the sigma-field generated by 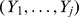 when 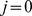 is 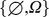; in particular, 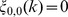, for all 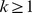.

The following asymptotic properties of 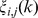 are useful in the remaining proofs.

**Lemma 14** *Assume that conditions (a)-(c) are satisfied and define* 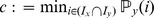 *. It follows that* 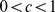 *and* 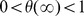. *Furthermore*

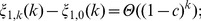
(32)

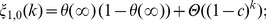
(33)

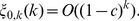
(34)


*Proof*. Observe that conditions (a)–(b) imply that 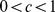. In addition, condition (b) implies that 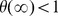, whereas condition (c) implies that 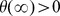.

Next, consider the set
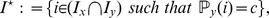
i.e. 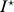 is the set of rarest colors in urn-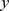 which are also in urn-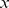. Also note that



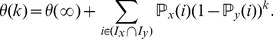
(35)As an intermediate step before showing (32), we prove that
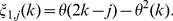
(36)


For this, first observe that
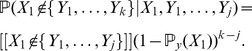



Hence
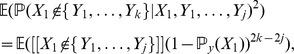


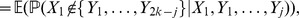


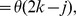
from which (36) now easily follows.

To show (32) note that (36) implies
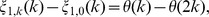


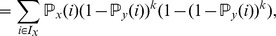


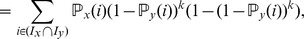


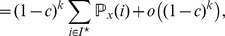
which establishes (32).

Now note that
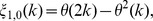


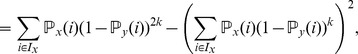


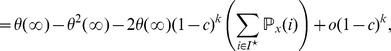
which establishes (33).

Next we show (34), which we note gives more precise information than (27). Consider the random variable 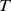 defined as the smallest 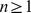 such that 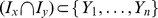. We may bound the probability of 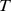 being large by 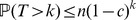, where 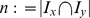 is finite because of condition (a). On the other hand, note that
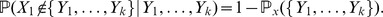



Define 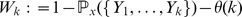 and observe that, over the event 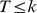, 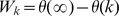. Since 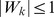, we obtain that
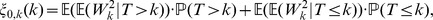


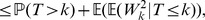


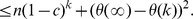



The identity in equation (34) is now a direct consequence of (35).

Our next goal is to show Theorems 4 and 5. To do so we rely on the method of projection by Grams and Serfling [17]. This approach approximates 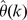 by the random variable
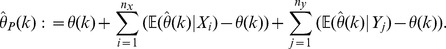



The projection is the best approximation in terms of mean squared error to 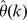 that is a linear combination of individual functions of each datapoint.

Under the stated conditions, 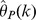 is the sum of two independent sums of non-degenerate i.i.d. random variables and therefore satisfies the hypotheses of the classical central limit theorem. The variance of the projection is easier to analyze and estimate than the 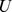-statistic directly, which is relevant in establishing consistency for the jackknife estimation of variance.

Let
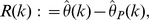
be the remainder of 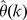 that is not accounted for by its projection. When 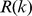 is small relative to 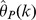, 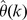 is mostly explained by 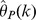 in relative terms.

The next lemma summarizes results about the asymptotic properties of 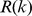, particularly with relation to the scale of 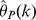 as given by its variance.

**Lemma 15** *We have that*

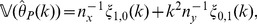
(37)

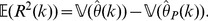
(38)


*Under assumptions (a)-(c), for a fixed* 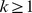, *we have that*

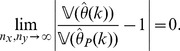
(39)


*Furthermore, under assumptions (a)–(d) we have that*

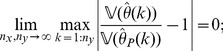
(40)



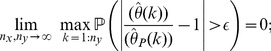
(41)

*for all* 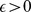.

*Proof*. A direct calculation from the form given in (16) gives that
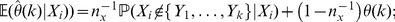
(42)

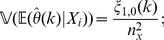


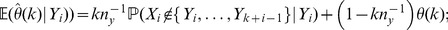
(43)

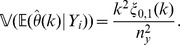



As 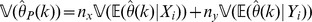, (37) follows.

To show (38), first observe that
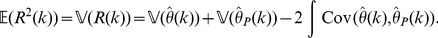
(44)


Next, using the definition of the projection, we obtain that
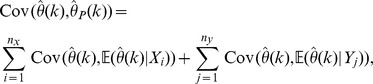


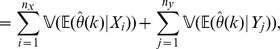


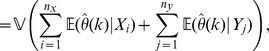


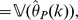
from which (38) follows, due to the identity in (44). Note that the last identity implies that 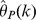 and 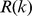 are uncorrelated.

Before continuing, we note that (41) is a direct consequence of (38), (40) and Chebyshev's inequality [22]. To complete the proof of the lemma all reduces therefore to show (41) under conditions (a)–(d). Indeed, if 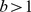 and we let 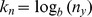 then due to the identities in (22) and (37) and Lemma 12, we obtain under (a)–(c) that
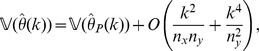
uniformly for all 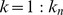, as 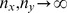. Since 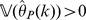 for all 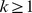, we have thus shown (39). Furthermore, note that 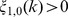 for all 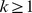; in particular, due to (33) and conditions (a)–(d), we can assert that 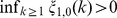. Since 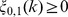, the above identity together with the one in (37) let us conclude that



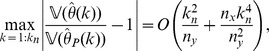
as 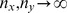. Because of condition (d), the big-O term above tends to 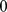. As a result:



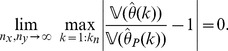
(45)On the other hand, (38) implies that 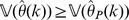. Hence, using (19) and (25) to bound from above the variance of the U-statistic, we obtain:
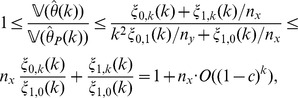
as 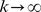, where for the last identity we have used (32) and (34). Since 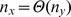, it follows from the above identity that



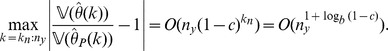



In particular, if the base-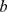 in the logarithm is selected to satisfy that 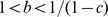, then
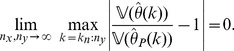
(46)


The identities in equation (45) and (46) show (41), which completes the proof of the lemma.

### Proof of Theorem 5

For a fixed 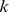, note that 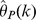 is the sum of two independent sums of non-degenerate i.i.d. random variables and thus,
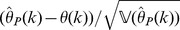
is asymptotically a standard Normal random variable as 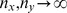 by the classical Central Limit Theorem. We would like to show however that this convergence also applies if we let 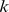 vary with 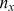 and 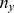. We do so using the Berry-Esseen inequality [23]. Motivated by this we define the random variables



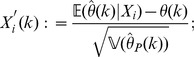


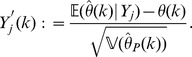



Note that 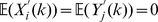, and that
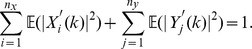



We need to show that
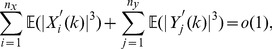
(47)uniformly for 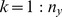, as 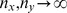.

Note that from (42) and (43),
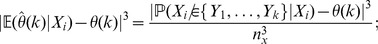


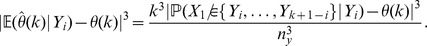



Let
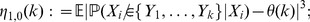


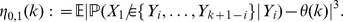



It follows from (37) that


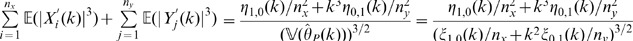
But note that 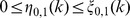. Since, according to Lemma 14, 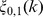 decreases exponentially fast, we obtain
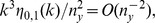
uniformly for all 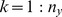, as 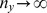. On the other hand, 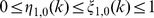. Furthermore, (33) implies that 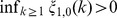. Since 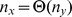, for some finite constant 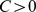 we find that



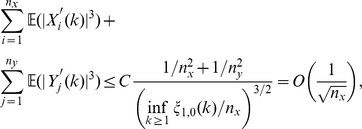
which shows (47).

The above establishes convergence in distribution of 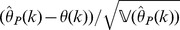 to a standard normal random variable uniformly for 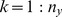, as 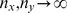. The end of the proof is an adaptation of the proof of Slutsky's Theorem [21]. Indeed, note that
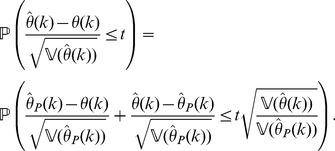
(48)


From this identity, it follows for any fixed 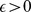 that


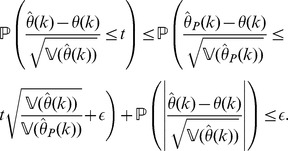


The first term on the right-hand side of the above inequality can be made as close to 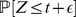 as wanted, uniformly for 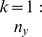, as 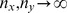, because of (40). On the other hand, the second term tends to 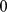 uniformly for 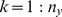 because of (41). Letting , shows that
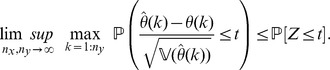



Similarly, using (48), we have:


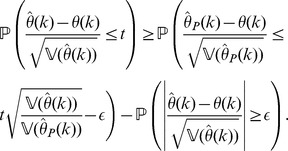


and a similar argument as before shows now that



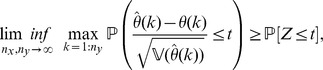
which completes the proof of the theorem.

We finally show Theorem 4, for which we first show the following result.

**Lemma 16** *Let* 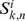 *be the set of one-to-one functions from* 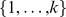 *into* 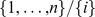 *. Consider the kernel* 
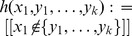
 *, and define*

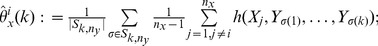
(49)

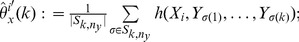
(50)

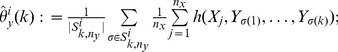
(51)

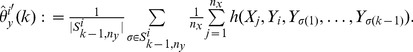
(52)
*Then, for each* 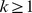 *and* 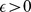,


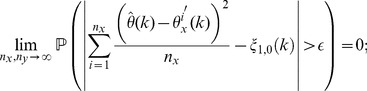
(53)


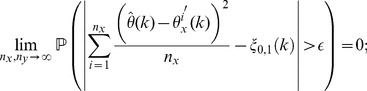
(54)

*Proof*. Fix 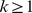. We first use a result by Sen [24] to show that, for each 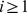:
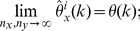
(55)

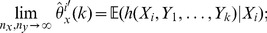
(56)

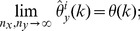
(57)

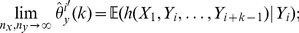
(58)


in an almost sure sense. Indeed, assume without loss of generality that 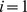. As the kernel functions found in (49) and (51) are bounded, the hypotheses of Theorem 1 in [24] are satisfied, from which (55) and (57) are immediate. Similarly, because 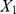 and 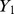 are discrete random variables, (56) and (58) also follow from [24].

Define
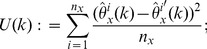
(59)

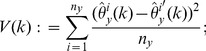
(60)and observe that



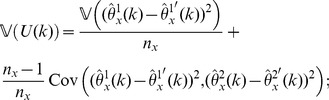


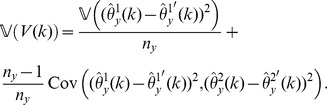



Furthermore, due to (55)–(58), we have that
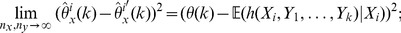
(61)

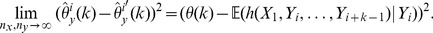
(62)


But note that, for 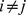, 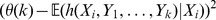 and 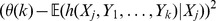 are independent and hence uncorrelated. Similarly, the random variables 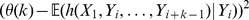 and 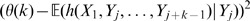 are independent. Since 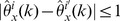 and 
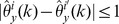
, it follows from (61) and (62), and the Bounded Convergence Theorem [22] that
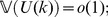
(63)

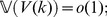
(64)as 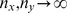.

Finally, by (30) and (31) it follows that
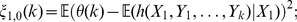


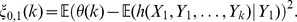



In particular, again by the Bounded Convergence Theorem, we have that 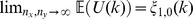 and 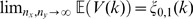. Since
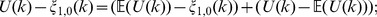


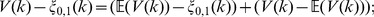
the lemma is now a direct consequence of (63) and (64), and Theorem 1.5.4 of Durrett [22].

### Proof of Theorem 4

Fix 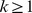. Using (16) we have that
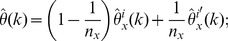


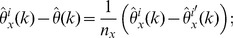


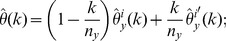


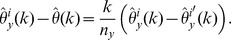



It follows by (28) and (29) that
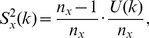
(65)

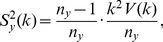
(66)where 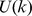 and 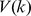 are as in (59) and (60), respectively. Furthermore, observe that



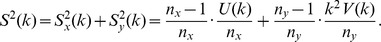



In particular, due to (37), we obtain that
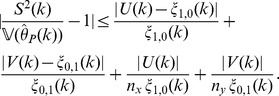



By Lemma 16, 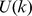 converges in probability to 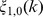, while similarly 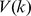 converges in probability to 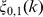; in particular, the first two terms on the right-hand side of the inequality converge to 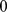 in probability. Since 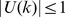 and 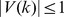, the same can be said about the last two terms of the inequality. Consequently, 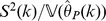 converges to 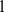 in probability, as 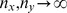. As stated in (39), however, conditions (a)–(c) imply that 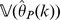 and 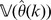 are asymptotically equivalent as 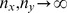, from which the theorem follows.

## Supporting Information

File S1 **Summary Metadata related to** **Table 1** **(tab-limited text file).**
(TXT)

File S2 **OTU table related to** **Table 2** **and** **Figures 1** **,** **2** **,** **3** **, and** **4** **(tab-limited text file).**
(TXT)
