# Correction: Estimation of Distribution Overlap of Urn Models

**DOI:** 10.1371/annotation/cd4610af-3595-4ea2-9409-71770712f2a9

**Published:** 2014-01-22

**Authors:** Jerrad Hampton, Manuel E. Lladser

Multiple errors occurred during the typesetting of this article; the publisher apologizes for these errors. In addition, the two-column format of PLOS ONE PDFs and typographic alignment of the PDF and online versions prevent some equations from being formatted correctly. Please view an alternative, correct version of the article here: http://www.plosone.org/corrections/pone.0042368.001.cn.pdf

